# Periodontitis and Cancer: Beyond the Boundaries of Oral Cavity

**DOI:** 10.3390/cancers15061736

**Published:** 2023-03-13

**Authors:** Alessandra Amato

**Affiliations:** Department of Neuroscience, Reproductive Science and Dentistry, University of Naples Federico II, 80138 Naples, Italy; aaleamato@gmail.com

Oral squamous cell carcinoma (OSCC) is the 16th most common cancer and the 15th leading cause of death worldwide, with an incidence of 4 cases per 100,000 people [1]. The main risk factors for OSCC are smoking and excessive alcohol consumption, associated with approximately 75% of cases [2]. Other risk factors include irritation of the mucosa due to trauma caused by dentures and rough tooth surfaces, poor nutrition, some chronic infections caused by specific microorganisms [2], such as Human Papillomavirus [3], Epstein–Barr virus [4], and Candida Albicans [5], and poor oral hygiene. The association between poor oral hygiene and oral cancer has been suspected for decades [6]. In recent years, oral dysbiosis, altering the fine equilibrium among microorganisms coexisting in the oral cavity and forming a large ecological community, with oral [7,8] and systemic health [9] relapses [10], as well as periodontitis have been implied in oral carcinogenesis [6].

Periodontitis is a microbially associated inflammatory disease [11] that commonly occurs in adulthood [12] and is characterized by periodontal tissue destruction and alveolar bone loss [13,14,15], which can lead to tooth loss [16]. According to the Global Burden of Disease Study, periodontitis prevalence has increased by 57.3% from 1990 to 2010 and currently represents the sixth most common disease worldwide, and affects about 743 million people, accounting for the 11.2% of the total world population [17], 10% of whom with severe periodontitis [18].

The association between periodontitis and OSCC may rely on periodontal and interrelated systemic inflammation, as hypothesized for several systemic inflammatory and degenerative diseases, including atherosclerosis, Alzheimer’s disease, age-related macular degeneration, and chronic bowel disease [19,20]. Indeed, chronic inflammatory processes were first linked to the development of cancer by Virchow in the 19th century. To date, 15–20% of human cancers are estimated to originate from an inflammatory process that promotes cell proliferation and malignant transformation [21]. Specifically, in animal models of periodontal biofilm-induced oral carcinogenesis, a higher incidence of epithelial dysplasia and oral carcinoma was found than in tobacco surrogate-induced carcinogenesis; a larger tumor size and a more marked reduction in the protective mechanisms of epithelial cell differentiation were also observed [6]. Moreover, more severe forms of periodontitis were correlated with a higher incidence of OSCC [22]. Most studies [23,24,25] specifically investigated the putative indirect oncogenic role of *Porphyromonas gingivalis* [23] in the initiation and development of OSCC [24,25,26].

Furthermore, oral dysbiosis underlying periodontitis has been associated with several malignancies [7] including lung [8], prostate [9], colon [10], pancreas [7], breast [27], head and neck [28], and oral cavity cancers [29,30]. The oral and periodontal microbiome could promote carcinogenesis at extraoral sites through systemic inflammation, indirect long-distance action of virulence factors from oral microbiota, direct translocation of microorganisms through the bloodstream, oropharyngeal, and respiratory tracts, or by influencing the response to treatments through interaction with the host immune response [31].

Periodontitis, especially in moderate or severe cases, has been associated with an increased risk of dying from cancer of the oral and digestive tract [26]. In addition, periodontitis has been reported to be an independent risk factor for the occurrence of complications in cancer patients undergoing gastrointestinal surgery for cancer [32]. In addition, the finding of an association between elevated serum IgG for *Porphyromonas gingivalis* and higher mortality for orodigestive cancers in both periodontitis and periodontally healthy individuals suggests a possible specific role of periodontal bacteria in the development of extraoral oncogenesis, independent of the presence of periodontitis [26].

Further studies are needed to understand the potential role of periodontitis, particularly some periodontal pathogens, in developing OSCC and extraoral cancers and the impact of periodontitis on cancer progression and patient outcomes for the multidisciplinary management of cancer patients with periodontitis [33,34].
